# Should I stay or should I go?

**DOI:** 10.7554/eLife.67417

**Published:** 2021-03-16

**Authors:** Mirjam Kretzschmar, Johannes Müller

**Affiliations:** 1 University Medical Center Utrecht, Utrecht UniversityUtrechtNetherlands; 2 Department of Mathematics, Technical University of MunichGarchingGermany; 3 Institute for Computational Biology, Helmholtz Center MunichNeuherbergGermany

**Keywords:** COVID-19, quarantine, SARS-CoV-2, epidemic containment, contact tracing, Human

## Abstract

Analysing the characteristics of the SARS-CoV-2 virus makes it possible to estimate the length of quarantine that reduces the impact on society and the economy, while minimising infections.

**Related research article** Ashcroft P, Lehtinen S, Angst DC, Low N, Bonhoeffer S. 2021. Quantifying the impact of quarantine duration on COVID-19 transmission. *eLife* **10**:e63704. doi: 10.7554/eLife.63704

The COVID-19 pandemic started just over a year ago, so there is a good chance that you have been in quarantine because you or one of your family, friends or colleagues tested positive for SARS-CoV-2. But how long should a person stay in quarantine before they can safely mix with others without posing a threat? Many countries implemented a 14 day quarantine period during the first wave of the pandemic, but it turned out that adherence to quarantine declined towards the end of this period (CDC, 2021; ECDC, 2020; Quilty et al., 2021; Steens et al., 2020). In many cases, this was because people could not afford to miss work for such a long time (Wright et al., 2020). If large numbers of people need to quarantine, this will impact productivity and be costly for the economy. At the same time, it is not clear that longer quarantines actually prevent many new infections. Because of this, many countries shortened their quarantines to ten days, and some allow release even earlier if individuals test negative before that time.

But, what is the optimal duration of quarantine that still ensures an effective control of SARS-CoV-2 transmission, while minimizing the individual and societal impact? Now, in eLife, Peter Ashcroft (ETH Zurich), Sebastian Bonhoeffer (ETH) and colleagues – Sonja Lehtinen (ETH), Daniel Angst (ETH) and Nicola Low (University of Bern) – report how they have used mathematical modelling to address this question (Ashcroft et al., 2021).

Based on estimated distributions of the time between a person getting infected and them infecting another person with COVID-19, the incubation period, and the infectivity of the virus, Ashcroft et al. quantified the impact of isolation and quarantine on onward transmission for index cases (the first identified case within a cluster) and their contacts. Index cases are identified through testing either when the individual develops symptoms, or when they return from travel from a country with high risk and get tested regardless of symptoms on entering their home country.

In the first case, knowing the distribution of incubation periods provides information about the possible time of infection and, therefore, the length of time an index case has had to infect others. For travellers, this information is less precise because it is harder to determine when they were infected, which will depend on the duration of travel and on how likely they are to have been exposed to infectious people in the country they travelled to. The analysis by Ashcroft et al. relies on estimating what proportion of onward transmissions could be prevented by various quarantine strategies.

At this point, Ashcroft et al. are faced with some arbitrariness in how to deal with optimizing a quarantine strategy that has several objectives (Denysiuk et al., 2015). On the one hand, reducing the spread of infection (the longer the quarantine is, the fewer onward infections), on the other, minimizing the societal and psychological consequences of quarantine. Ashcroft et al. manage this problem by using a utility function that measures the proportion of transmissions prevented per extra day of quarantine, merging the two aspects that need to be optimized. However, this is just one of several possible ways to handle the task, and it is not clear that it is the best approach.

Furthermore, Ashcroft et al. may be underestimating the effect of quarantine, since they are only counting the number of prevented direct infections, but not the people these prevented infectees would otherwise be infecting. In regions where the virus is highly prevalent, these infection chains might overlap, and affect the net number of prevented cases. Even if the utility ratio were the best approach to optimize a quarantine strategy, this ratio will depend on the state of the epidemic.

Ashcroft et al.’s results have implications for how to best balance public health needs with societal interests of reducing the costs of quarantine. First, the delay between exposure of an index case and isolation and quarantine of their contacts should be minimized in order to prevent as much onward transmission as possible. Second, quarantine periods of less than five days after exposure are not effective, but effectiveness hardly increases after ten days of quarantine. Between these bounds, the optimal quarantine duration lies between six and eight days, with contacts being released if they test negative after that time (Figure 1). This strategy would decrease the load on society by reducing the number of people in quarantine at the same time, and likely lead to higher adherence to quarantine measures. To further reduce the probability of transmission after release from quarantine, the timing of testing should also be optimized (Wells et al., 2021).

**Figure 1.**
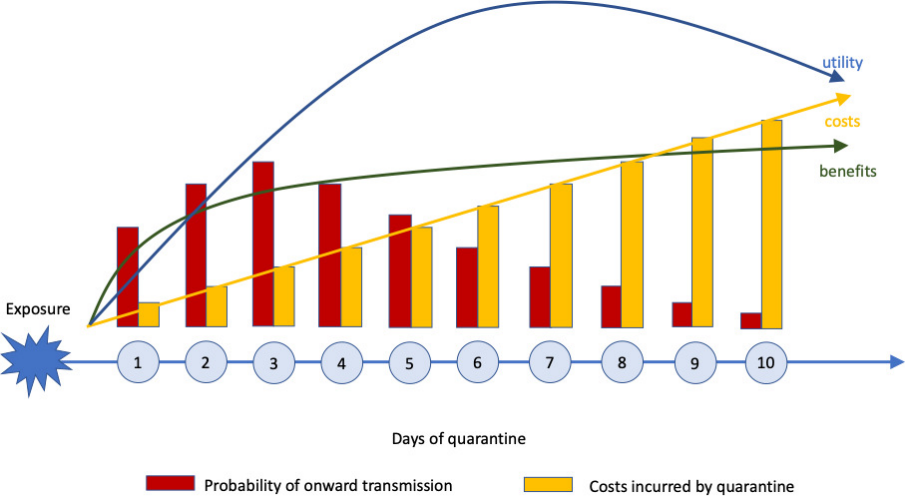
Costs versus benefits of quarantine depending on time. The costs of quarantine (yellow bars and arrow) increase steadily with time, while the benefits (green arrow) – measured as number of onward transmissions (red bars) prevented – increase steeply at first, and then flatten. Ashcroft et al. estimate that balance between costs and benefits – known as the utility (blue arrow) – increases at first, reaching a peak after 6–8 days, and then decreases.

The analysis reported by Ashcroft et al. assumes that quarantine is complete in the sense that as long as a person is in quarantine, onward transmission is prevented completely. In practice, this will often not be the case, as people live in households with others, where they may not be able to avoid contact and transmission. Therefore, quarantine needs to be extended to the people who live with the contacts of an infected person, meaning that the costs incurred by quarantine depend on household size and other factors that determine how well quarantine can be implemented in practice. There is no question, however, that a test-and-release strategy, preferably using rapid tests with high sensitivity, can help to combine control of the pandemic with societal acceptance of the measure.

These results emphasize the impact of implementing widespread, low-threshold testing strategies. Additionally, they underline the importance of clearly communicating that people do not need to stay in quarantine longer than necessary, but that there is an evidence-based strategy behind their having to stay home (Smith et al., 2020; Webster et al., 2020). It will be possible to go out again, but not too early. The virus can tell us when the time has come.
